# Recurrent transitory attacks with cytotoxic edema could benefit from thrombolysis

**DOI:** 10.1186/s12245-024-00601-9

**Published:** 2024-03-05

**Authors:** Josef Finsterer, Sounira Mehri

**Affiliations:** 1 Neurology Dpt., Neurology and Neurophysiology Centre, Postfach 20, Vienna, 1180 Austria; 2 Biochemistry Laboratory, LR12ES05 “Nutrition-Functional Foods and Vascular Health”, Faculty of Medicine, Monastir, Tunisia

**Keywords:** Stroke, Transitory ischemic attack, Thrombolysis, Cerebral MRI, Atrial fibrillation

We read with interest the article by Tan et al. about a 65-year-old male with ten transitory ischemic attacks (TIAs) within three hours, clinically manifesting as dysarthria and right-sided hemiparesis, each lasting approximately 10 min [1]. ECG showed newly diagnosed atrial fibrillation (AF). Cerebral computed tomography (CCT) showed an old left, lacunar striato-capsular infarct and computed tomography angiography (CTA) showed only mild atherosclerosis [1]. Magnetic resonance imaging (MRI) showed a small acute infarct in the left corona radiata three hours after onset [1]. The patient recovered completely under therapy initially with acetyl-salicylic acid (ASS, 300 mg), clopidogrel (300 mg), and “aggressive hydration”, which was replaced by apixaban 10 mg/d three days after onset [1]. The study is impressive, but several points require discussion.

The major limitation of the study is that the patient did not have capsular warning syndrome (CWS) [1]. “Capsule” refers to the internal capsule. However, MRI showed an ischemic lesion in the left corona radiata, which is distinct from the internal capsule. Therefore, the diagnosis of CWS is not justified. A second argument against CWS is that dysarthria and hemiparesis are not necessarily due to an internal capsule lesion. They also coincide with the lesion site found on the MRI.

A second limitation is that the patient did not undergo thrombolysis. The patient scored nine points on the national institute of health stroke score (NIHSS), had an ischemic stroke on MRI, and was within the time window for thrombolysis. He had no contraindications. This constellation is a clear indication for thrombolysis. There are no international guidelines recommending rTPA administration only for TIAs that last longer than 10 min. “Aggressive hydration” is not an approved treatment for TIAs or stroke. If national or institutional regulations allow other procedures, this should be reported.

The third limitation is that the patient has not already undergone an MRI upon arrival at the hospital. Even with a clinical presentation of recurrent TIAs, cytotoxic edema could have already occurred at the onset of the clinical deficits. It is possible that the indication for thrombolysis existed earlier than after the MRI. The earlier thrombolysis is carried out, the more effective it is. If institutional equipment does not permit acute MRI, multimodal CT should be performed.

A fourth limitation is that echocardiography was not performed. To determine whether AF was valvular or non-valvular and to determine whether valvular, atrial or ventricular thrombus formation was present, performing echocardiography, preferentially transesophageal echocardiography, is critical. It would also have been helpful to know creatine-kinase, troponin, pro-natriuretic peptide (proBNP) levels to determine whether non-ST-elevation myocardial infarction (NSTEMI), Takotsubo syndrome, or heart failure was present. If real world conditions delay performing the echocardiography, this may place the patient in additional danger and may delay causal therapy.

In conclusion, the excellent study has limitations that should be addressed before drawing final conclusions. Clarifying the weaknesses would strengthen the conclusions and could improve the study.

## Data Availability

all data are available from the corresponding author.
